# Implementation of a quality management system for decentralized manufacturing of cell and gene therapy products - technical and regulatory considerations

**DOI:** 10.3389/fmed.2025.1591751

**Published:** 2025-08-05

**Authors:** Heiko von der Leyen, Julio Delgado, Chaya Mazouz, Michael Schmitt, Vered Caplan

**Affiliations:** ^1^Orgenesis Inc., Germantown, MD, United States; ^2^Oncoimmunotherapy Unit, Hospital Clínic Barcelona (HCB), IDIBAPS Biomedical Research Institute, University of Barcelona, Barcelona, Spain; ^3^Department of Hematology, Cellular Immunotherapy, University Hospital Heidelberg, Heidelberg, Germany

**Keywords:** decentralized manufacturing, cell and gene therapy, control site, point of care platform, GMP compliance, quality management system, regulatory oversight

## Abstract

Decentralized manufacturing has emerged as a promising approach to improve the accessibility and scalability of cell and gene therapy products, particularly for autologous treatments. This paper proposes a comprehensive Quality Management System (QMS) framework tailored to decentralized cell therapy manufacturing, integrating current Good Manufacturing Practice (cGMP) principles and regulatory oversight through a centralized *Control Site* model. The *Control Site* serves as the regulatory nexus, maintaining POCare Master Files and ensuring consistency across multiple decentralized manufacturing sites. Decentralized manufacturing has the potential to facilitate accessibility for cell and gene therapies. The proposed model leverages automated, closed-system technologies to minimize process variability and hardware deviations, thereby enhancing product quality and regulatory compliance. The *Control Site* holds functional roles like primary focus point for interaction with regulatory agencies, provision of quality assurance, qualified person (QP) and oversight systems. It also maintains the POCare Master File for the individual POCare GMP manufacturing sites. A standardized GMP manufacturing platform (e.g., deployable as prefabricated units allowing quick expansion) and an overarching training platform should guarantee quality standards. Key regulatory expectations will be discussed, e.g., the demonstration of consistency and comparability, the central role of QP (as proposed in the context of the European Commission’s Pharma strategy), and the C*ontrol Site* as single point of contact for competent authorities. This approach aims to streamline cell therapy production at or near point of care, supporting rapid and cost-effective clinical implementation.

## Introduction

Currently, both allogenic and autologous therapies rely on centralized manufacturing. This may continue to be viable for allogenic therapies (1), which can be manufactured in bulk and stored as cryopreserved cell products. However, given complex logistics and time constraints associated with autologous cell therapies a decentralized manufacturing approach may represent an approach for better availability and affordability of cell therapy. Decentralized manufacturing of autologous therapies could occur in one of two settings: (i) regional facilities managed by industrial developers or contract manufacturing organizations (CMO), or (ii) across certified treatment delivery centers (e.g., academic health centers) close to the patient’s bedside. Thus, decentralized or agile manufacturing (2) describes manufacturing strategies employed within a network of manufacturing sites encompassing manufacturing and organizational methodologies as well as enhancing supply chain and business flexibility. Point of care (POCare) describes the location (close to patients) where a product is being manufactured. Terminology for POCare or decentralized manufacturing is not standardized, and a range of often-overlapping terms including distributed manufacturing, redistributed manufacturing or GMP-in-a-box are sometimes used (Table 1).

**TABLE 1:** Definitions.

Term	Definition
**Treatment**	
Point of care (PoCare) Place of care	Site at which patient treatment is conducted
**Manufacturing**	
– Decentralized (3) – Agile (2) – Distributed (13) – Redistributed (5)	Product manufacturing at multiple sites under central management
GMP-in-a-box (24)	Systems enable manufacturing in lower-grade cleanrooms

Following, we will describe a quality management framework for the realization of decentralized manufacturing. Transition from standard centralized manufacturing to a decentralized approach requires rethinking of some established Good Manufacturing Practice (GMP) principles. However, the proposed regulatory set up does not imply that GMP requirements are being undermined or minimized when applied for decentralized manufacturing. Current centralized manufacturing processes, shipment and respective cryopreservation are time consuming and may delay the application of the cell therapy to patients in need (3). For optimal implementation of decentralized manufacturing closed-system manufacturing that minimizes the infrastructure requirements at treatment facilities should be implemented maintaining compliance with regulatory requirements and quality standards (1, 2). The transition from centralized to decentralized manufacturing is a significant process, thus a comprehensive Quality Management System (QMS) is required which must be based on current GMP principles.

## The need for decentralized manufacturing

In the field of cell therapy, the intrinsic variability of product starting materials obtained from patients, the subsequent variability of the therapeutic products, and complexity of shipments represent peculiar situations different from standard pharmaceutical drug production. Accordingly, regulatory strategies have been evolved for unique patient-specific cell therapies that take these differences into account. Currently, the pharmaceutical industry has relied on a centralized model of cell therapy product manufacturing. This approach poses significant challenges in terms of manufacturing capacity and the timely delivery of patient-specific cell therapy products on a large scale.

As a solution to these challenges decentralized manufacturing is being considered, that is, “*technology, systems and strategies that change the economics and organization of manufacturing, particularly with regard to location and scale*” (4). According to the Redistributed Manufacturing in Healthcare Network (RiHN) consortium, motives for redistribution of manufacturing include (i) cost reduction through terminal customization, (ii) just-in-time delivery (particularly of fresh products with short stability), (iii) management of capacity by distributed production through scale-out, (iv) reducing up-front capital cost, and (v) building small production units in an incremental response to increased demand (5).

A survey-based study by BioPlan Associates indicates that cell and gene therapy manufacturing is experiencing a serious manufacturing “capacity crunch,” particularly at commercial level (6). A shortage of cell and gene therapy manufacturing capacity is being estimated at 500%. Although approximately 90% of cell and gene therapy developers would prefer to use CMOs (7), the current CMO capacity is not sufficient to meet this need. The lead time for CMOs to begin cell and gene therapy projects currently exceeds 18 months (8). Many CMOs are expanding their CGT manufacturing capacity, but setting up large-scale extension buildings can often take years until they are fully operational. As a result, many CGT developers are seeking alternatives to expand their manufacturing capacity, and regulatory authorities have initiated discussions on new regulatory frameworks enabling decentralized manufacturing.

Consequently, major changes in product manufacturing paradigms may be realized in the field of cell and gene therapy (CGT). New technological solutions like closed-system automated manufacturing, digitization, and rapidly deployable manufacturing units will enable decentralized manufacturing. To ensure GMP quality adherence a new Quality Management System (QMS) must support decentralized manufacturing. Competent authorities like MHRA, FDA and EMA already consider decentralized manufacturing as an option for CGT manufacturing.

### Point of care and decentralized manufacturing – MHRA view

The United Kingdom (UK) is the first country to introduce a tailored framework for the regulation of innovative products manufactured at the point of care where a patient receives care (9). Patient-specific medicines with very short shelf lives can more easily be made in or near a hospital setting and delivered to the patients who need them more quickly. The new framework will reduce or eliminate regulatory barriers to innovative manufacturing, while ensuring that advanced therapy products made in these novel ways have the same assurances of quality, safety, and efficacy as those for conventional medicinal products (9). UK’s competent authority, the Medicines and Healthcare products Regulatory Agency (MHRA), describes several regulatory framework features of products manufactured at POCare which make progress for such products difficult and in some cases almost impossible:

• Short shelf-life
• Manufacturing at a large number of sites
• Intermittent nature of manufacturing
• Novel and wide range of manufacturing location types
• Wide range of product types.

In its new regulatory framework MHRA has created two new licenses for medicinal products: “manufacturer’s license (modular manufacturing, MM)” and “manufacturer’s license (Point of Care, POC).” As holders of these manufacturing licenses a “control site” will be established meaning that it has responsibility to supervise decentralized manufacturing (9). The POCare framework is new, however, it will be based on current regulatory control systems like inspection, clinical trials, marketing authorizations and pharmacovigilance. It will enlarge the spectrum of products and supply models to meet widening therapeutic needs (10). In January 2025, MHRA implemented the change of the existing drug regulations enabling decentralized manufacturing (11).

### Point of care manufacturing – FDA view

In 2021, the US National Academies of Sciences, Engineering, and Medicine issued a report titled “Innovation in Pharmaceutical Manufacturing on the Horizon: Technical Challenges, Regulatory Issues, and Recommendations” noting potential innovations in integrated, flexible, and distributed manufacturing (12). The report also states that these potential innovations would include modular approaches to streamlining drug development and production. This includes the deployment and use of highly portable manufacturing units. Such highly portable units could enable localized POCare manufacturing and precision medicine (12).

FDA’s Center for Drug Evaluation and Research (CDER) initiated, through the Emerging Technology Program, the Framework for Regulatory Advanced Manufacturing Evaluation (FRAME). Herein, Distributed Manufacturing is proposed as a platform with manufacturing units that can be deployed to multiple locations enabling POCare manufacturing in proximity to patient care, for example, at healthcare facilities (13). At the 2022 Cell and Gene Therapy Meeting on the Mesa, the Center for Biologics Evaluation and Research director Peter Marks shared how the FDA is working toward advancing CGT development, manufacturing, and approval, and mentioned that “*automated manufacturing could be another solution to help lower the costs of production which are significantly higher for cell and gene therapies than for other more established drug types*” (14).

In its draft guidance on the development of CAR-T cell products the FDA discusses that the same type of CAR-T cells may be manufactured at several facilities (15). In their opinion, manufacturing at multiple sites may shorten the timeline from cellular starting material collection to administration for autologous products. However, differences between manufacturing facilities may contribute to product variability. Thus, the sponsor should demonstrate that a comparable product is manufactured at each location. Sponsors are supposed to also demonstrate that analytical methods are comparable across the different sites, if applicable (15). Comparability is crucial for decentralized manufacturing and will be further discussed in more detail below.

### Point of care manufacturing – EMA view

In 2020, the European Medicines Agency (EMA) and the Heads of Medicines Agencies (HMA) published a network strategy 2025 focusing on the protection of public health “at a time of rapid change” (16). Six priority areas for the network were identified, v.a. the availability and accessibility of medicines and supply chain challenges. This report states that “*proponents contend can ultimately be combined in a closed, easy-to-operate, tabletop-sized machine with integrated production and purification that could be used in for example a* hospital *pharmacy or operating theater (decentralized manufacturing) or even mobile clinics to provide customized products designed to address the needs of an individual patient*” (16). Implementing a POCare manufacturing approach in a clinical study, Maschan et al. reported robust safety and clinical responses in patients with relapsed/refractory B cell malignancies treated with place-of-care manufactured anti-CD19 CAR-T cells produced at two different locations (17). In this study “place-of-care manufacturing” was defined as near the point of patient treatment allowing cell products to be produced and infused without the need for cryopreservation (17).

The Guideline on Good Manufacturing Practice specific to Advanced Therapy Medicinal Products (published in 2017) describes a batch release process in cases of decentralized manufacturing [EudraLex Vol 4, C(2017)/7694] (18). It says: “*There may be cases where manufacturing of the ATMP needs to take place in sites close to the patient (e.g., ATMPs with short shelf-life, clinical advantage of using fresh cells as opposed to freezing the starting materials/finished product, etc.). In such cases, manufacturing of the ATMPs may need to be decentralized to multiple sites so as to reach to patients across the EU. This scenario may occur both in the context of authorized ATMPs as well as in the context of investigational ATMPs.”* Specifically, the following points are being made:

• Central site in EU for oversight
• A qualified person (QP) established in the EU has the ultimate responsibility
• Remote data from decentralized manufacturing sites can be transmitted by qualified personnel to the QP.

European Medicines Agency’s newly established Quality Innovation Expert Group (QIG) supports innovative approaches for the development, manufacture, and quality control of medicines for the benefit of patients in the European Union (EU). These include, but are not limited to, new technologies, digitalization, novel materials and novel devices, in line with the priorities highlighted in EMA’s Regulatory Science Strategy to 2025 (19). The role of QIG is to ensure that the European medicines regulatory network keeps pace with innovation, identifies and addresses gaps in the regulatory framework, and increases predictability for developers of innovative technologies. Considering that development and manufacturing medicines is global in nature, QIG also aims to establish close collaboration with international partners to facilitate global regulatory convergence (19).

Since innovative approaches for the development of manufacturing and quality control of medicines are becoming the new paradigm to be faced both from an industrial and regulatory perspective, features such as very short shelf-lives and highly personalized products like ATMPs may need a “decentralized” manufacture (local to the patient) at different locations, such as hospitals, pharmacies or even mobile units (16). According to QIG the current legislation does not sufficiently address the details associated with the decentralized paradigm in terms of supervision system of decentralized sites and lifecycle management (19).

In summary, MHRA, FDA and EMA all identified decentralized manufacturing as an important addition to providing medicinal products to patients in need. MHRA implemented the respective legislation in January 2025, FDA has included in its FRAME program the option for distributed (de-centralized) manufacturing employing multiple manufacturing units in proximity to healthcare facilities. EMA already described in EudraLex Vol. 4 (2017/7694) the case of decentralized manufacturing of ATMPs at multiple sites. In contrast to MHRA, FDA and EMA have not yet published specific guidelines for decentralized manufacturing. Based on the above-described facts we here propose a model employing decentralized manufacturing with a central QMS and “control site” oversight enabling cost reductions, in particular in conjunction with fully automated manufacturing and digitization solutions (20).

## Realization of decentralized manufacturing at point of care

Platforms for POCare manufacturing should enable a high-quality, standardized, efficient, and scalable pathway for production and distribution of advanced therapies. Hereby, affordable availability of cell therapy products to many patients would be promoted at their treatment sites. Ideally, such a POCare platform would be designed around a harmonized, decentralized manufacturing model based on a dedicated network of clinical sites, and wherever possible, by leveraging proprietary disruptive manufacturing infrastructures and technologies, thus enabling *agile manufacturing* (2). We refer to this as the POCare Platform which includes the clinical sites as POCare Centers.

The POCare Platform consists of a POCare network with enabling technologies and infrastructure elements:

### POCare network

• Local decentralization: POCare centers are established in collaboration with regional partners, based on the capacity needs of nearby hospitals (sites), forming the POCare network. The POCare network can bring together physicians, CGT industry partners, research institutes, medical centers and hospitals, regionally or even worldwide.
• Global harmonization: The POCare platform overcomes conventional processing challenges by enabling high quality standards, scalable, onsite, aseptic processing of CGTs within POCare centers, and servicing local hospitals. The network structure is supported and connected by adhering to current GMP practices and standards to meet the highest quality standards, and an overarching Quality Management System (QMS) enabling harmonization and central oversight.
• Training program: the purpose of the training program is to ensure that training is delivered in a manner and frequency to ensure the required level to perform good practice (GxP) activities. The training program should include at least:
  ∘ GxP training
  ∘ At least every year, a GxP refresh, including review of applicable regulations and recent regulatory requirement changes
  ∘ Global Policies and Quality Standards as appropriate
  ∘ Job-specific SOPs and working instructions as appropriate.

Disruptive manufacturing technologies are designed to harmonize and optimize decentralized manufacturing. They cover both the manufacturing environment requiring highest quality standards and the efficiency and scalability of the manufacturing process itself. Enabling technologies support availability, affordability and accessibility to reach the goal of efficient cell therapy for patient care at point of care.

### Enabling technologies

• Availability: developing and optimizing cell processing for cell and gene therapy that are designed to be produced in closed, automated technology systems, reducing the need for high grade cleanroom environments.
• Affordability: Decentralized manufacturing in closed systems eliminates complicated logistics and reduces manufacturing failure risk and the high cost of manual processing. Standardization and harmonization are feasible for automated closed systems which are customized for a given therapy and available as a total manufacturing solution that ensures consistent quality and supply.
• Accessibility: Mobile manufacturing environment solutions are available for rapid on-site deployment without the need for expensive infrastructure. A global collaborative POCare centers network can serve local leading hospitals and medical centers applying cell and gene therapy. The required automation provides an inherent distribution opportunity for existing and future therapies.

Decentralized production of therapies must be carried out in a strictly controlled and monitored environment in full accordance with GMP. International guidelines define the manufacturing quality framework, but disparities remain at local regulatory levels. However, to ensure scalability of the decentralized model but also harmonization of good manufacturing practices, it is necessary to operate in a standardized environment and infrastructure. According to EMA regulations cell therapy products like CAR-T cells manufactured at a decentralized site (under industry or academic sponsorship, respectively) or manufacturing under hospital exemption must comply with GMP standards (21). Manufacturing success does seem to be not different between products manufactured by industry vs. academia. Martinez et al. reported for the academic CAR-T19-BE-01 trial a 94% manufacturing success rate (20). This is in line with other CAR-T-cell products like the commercially produced tisa-cel in its application for R/R DLBCL (93%) (22). A recent academic study reported even a 100% GMP production success (23). Mobile, prefabricated and thus easily deployable manufacturing units can provide similar clean room infrastructures on a highly controlled basis and according to current GMP (cGMP) standards. Manufacturing variability of sites will be reduced to a minimum. To guarantee adherence to quality requirements the respective staff involved in manufacturing tasks need to be specifically trained in applying adequate SOPs. Furthermore, complete automation of the manufacturing process including automated in-process controls and digitalization will reduce hands-on work and thus manual labor-related failures (24).

## Comparability as basic requirement for decentralized manufacturing

It is the responsibility of the sponsor to qualify the manufacturing process and confirm that all regulations are being followed at each POCare manufacturing facility. The regulatory submission should clearly describe a quality oversight strategy for a decentralized manufacturing approach.

The regulatory submission should describe in detail how quality systems will ensure that each manufacturing facility is capable of consistent manufacture of a product with appropriate safety and quality attributes, according to cGMP and other applicable regulatory requirements. It must be demonstrated and established that a comparable product is being produced at each facility, and that comparable analytical assays are performed at each facility. Assessment of a product’s overall clinical safety and efficacy must rely on clinical data resulting from the administration of comparable products to patients.

Comparability studies will always be necessary when opening a new manufacturing site for a given process/medicinal product. Implementing automated manufacturing solutions together with digitalization will drastically reduce the costs for these quality assurance tasks. Providing similar automated manufacturing bioreactors at each manufacturing site will surely reduce manual labor related failures to a minimum. Lam et al. compared centralized vs decentralized manufacturing of a current standard CAR-T product and demonstrated that with anticipated increases of demand for cell therapy products the decentralized model becomes more comparable cost-wise, thereby having greater demand stress resilience, improved resource utilization and opportunities for economies of scale (25). The implementation of less labor intensive advanced automated production systems will further contribute to the cost savings by decentralized manufacturing. Reduced manufacturing costs may also contribute to wider distribution of cell therapy products to low-and middle-income countries. A study in India employing a CAR-T product reported production costs of only $ 15,000 (26).

A comparability exercise must be conducted stepwise, starting with the physico-chemical and biological properties of the product (27). This is based on analytical testing, e.g., routine batch analysis, in-process controls, process validation/evaluation data, characterization and stability studies, as applicable. The focus must be on manufacturing process steps most appropriate to detect a change which require an evaluation on all critical steps/in-process controls/materials of the manufacturing process downstream of the change. Any observed analytical difference should be evaluated in relation to its impact on product quality, safety and efficacy (28). EMA published detailed questions and answers regarding comparability considerations related to ATMPs (27). The goal of comparability exercises is to ascertain that CGT drug products (manufactured by each site) are highly similar in terms of quality as it may relate to the safety and effectiveness of the product (15). Information on data from comparability studies can be found on published summaries of biological license applications (FDA) and marketing authorization applications (EMA). Examples have been summarized by Cockroft and Wilson indicating the importance of comparability studies (28).

Comparability data should be generated based on a prospective comparability protocol, to assess the effect of different manufacturers on product quality. A comparability protocol should include vice altera the following topics (27, 29):

a) Description of the representative dataset to be used for the comparability study (i.e., direct side-by-side comparison with or without split of source material).
b) In case of autologous starting material, splitting the same source material should be considered to manufacture the lots for the comparability evaluation.
c) Quality attributes to be evaluated including description of the analytical methods used for evaluation of Critical Quality Attributes (CQAs).
d) Description of statistical methods for evaluating comparability, if appropriate.
e) Effect of the manufacturing location on the stability of the product or justification why additional stability evaluation is not needed.

Without providing adequate evidence of product comparability, the use of multiple manufacturing sites may create a risk that clinical outcome of a given therapy will not be consistent. As described in detail in the next chapter, the *Control Site* will be responsible for regulatory oversight to ensure that a comparable product is being produced at each facility, and that comparable bioreactors as well as analytical assays (including in process controls) are performed at each manufacturing site. Initially, POCare networks will probably be set up in specific geographical areas to cover the respective regulatory jurisdiction (e.g., FDA or EMA). Later, when harmonization within the International Council of Harmonization (ICH) process will be achieved, a world-wide acting POCare network could be established considering the regulatory requirements for comparability.

## Consolidated proposal for QMS regulation of decentralized manufacturing

A decentralized manufacturing platform has multiple facilities covering defined geographical areas, allowing products to be manufactured and distributed close to medical centers and patients. Thus, the time from harvesting the starting material (cells or tissue) to treatment of the patient could be shortened considerably. Although decentralized manufacturing at a POCare is a new concept, it is based on and linked to current regulatory tools, standards, and approaches including inspection and clinical trials. Regulatory challenges include questions of product variability and comparability manufactured at multiple sites (as described before) and regulatory compliance mechanisms for control and oversight of the manufactured product.

### POCare centers

Establishing regional POCare centers may serve as a comprehensive approach addressing the “capacity crunch” by providing similar manufacturing environments, while enabling flexible localization of processing units to minimize logistic complexity and to enable scale out. The POCare platform is supported by a regulatory methodology, based on present regulatory thinking. Adherence to GMP standards across the POCare network of facilities will be established by a *Control Site*, serving as the primary focus of regulatory interface.

### Quality management system (QMS) and control site

**FIGURE 1:**
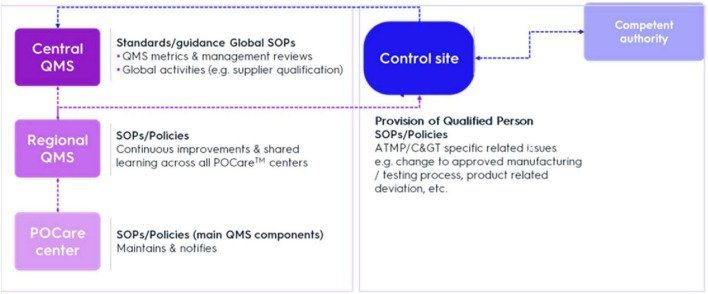
Mechanisms for control and oversight. QMS, quality management system; SOP, standard operation procedure.

To ensure regulatory compliance with decentralized manufacturing quality requirements, broad oversight of *POCare centers* needs to be established. The QMS comprises of an overarching system providing general standards for regional entities around the world. Regional QMS integrate region-specific requirements according to local authorities (e.g., EMA or FDA), thereby specifically ensuring global harmonization of quality. In addition, decentralized sites may initially have different local QMS. Thus, this approach must support limited release timeframes while taking into consideration inherent product and POCare variability. Creating uniform QMS and oversight initially will require upfront investment in harmonization of quality management processes but will result in increased efficiency and cost reduction. In general, *Central and Regional QMS* support GMP oversight, while the *Control Site* addresses CGT specific related issues, e.g., change of initially approved manufacturing/testing process or product related deviation. The *Control Site* is the primary focus of regulatory oversight and controls (Figure 1).

Central (or global) QMS (C-QMS) provides a set of standards/guidance documents setting main QMS elements for regional entities around the world, which are the quality entities in relation to the POCare centers. In addition, C-QMS establishes procedures addressing global operation-related quality activities, e.g., supplier qualification. Through the integration of a regional QMS (R-QMS) with C-QMS, quality comparability across the POCare platform and application of good practices (GxP) across all activities and throughout product lifecycle can be secured.

Set-up tasks and accountabilities can be summarized as follows:

I. Central (Global) QMS
  a. Standards
  b. Policies
  c. Auditing
  d. Supply chain
  e. GMP readiness

II. Regional QMS
  a. Regional quality oversight
  b. Overseeing several POCare sites
  c. Part of global organization
  d. Reinforce oversight
  e. Audits
  f. Process development and transfer

III. Control site
  a. Technology transfer oversight
  b. Interaction with competent authority
  c. Provision of qualified person (QP)
  d. Oversight in “day to day” activities
  e. Oversight of change of initially approved manufacturing/testing process or product related deviation
  f. POCare Master File for individual POCare GMP manufacturing sites

IV. POCare sites
  a. Receiving starting material
  b. Manufacturing
  c. Release
  d. Shipment

The QMS should be compliant with EU and FDA requirements and should be based on International Council for Harmonization (ICH), 21 CFR 11, as well as Pharmaceutical Inspection Convention/Pharmaceutical Inspection Co-operation Scheme (PIC/S) requirements. Based on geographical area, regional QMS should be compliant with C-QMS standards/guidance and applicable regional regulations and requirements. Regional Quality Assurance (QA) leaders should report to the central head of quality. Central (global) QA activities include among others assessment and establishment of new sites, decommissioning and removal of POCare sites, training, oversight of QMS, provision and control of manufacturing equipment, raw and starting materials and consumables (Table 2).

**TABLE 2:** Distribution of roles and responsibilities between central QMS and regional QMS.

Category	Major tasks	Global quality	Regional quality
**Set-up of manufacturing capabilities**			
	GMP POC site readiness	C	A
	Global QMS compliance	C	A
	Manufacturing license authorization	C	A
**Product/process characterization**			
	Product stability studies	I	I
	Master batch records and test methods (end of DEV stage)	C	A
	Establishment of product equivalence/comparability	I	A
	Continued process verification and consistency levels	I	I
	Product quality review	A	A
**GMP manufacturing capabilities**			
	Technology transfer to reference site (including tools and digital connectivity)	C	A
	Training	C	A
**Routine manufacturing**			
	Day to day manufacturing related activities	C	A
	Batch records and CoA review/approval	C	A
	Deviation/OOS/CAPA/ change/complaint management	C	A
**Regulatory submissions**			
	Ensure compliance to regulatory file/product license	I	I

RACI matrix R, responsible; A, accountable; C, consulted; I, informed; (36). DEV, development; OOS, out of specification; CoA, certificate of analysis.

### The control site role and responsibility

A “control team” will be designated comprising of CGT manufacturing/testing subject-matter experts (SME) as well as regulatory and quality experts. The SME acts as the main contact point for manufacturing/testing issues for a designated CGT product, preferably the SME is selected from the product development process experts (see below). “Control site” oversight and QMS are part of the quality agreement between sponsor and manufacturer (e.g., CMO). The “control site” is included in the quality agreement with the sponsor and represents the contact person for competent authorities. The “control site” should be established in a specific region enabling close interaction with local competent authorities, e.g., in conjunction with the reference center. As described above depending on the number of involved manufacturing sites, comparability studies will be implemented to qualify the manufacturing process and confirm that all regulations are being followed at each POCare manufacturing facility (see the previous chapter).

The *Control Site* holds the following roles:

• Primary focus point for interaction with regulatory agencies and sponsors.
• Provision of QA and qualified person (QP) oversight.
• Product release according to current GMP requirements.
• Systems implemented to capture and provide information from R-QMS to the control site, traceability information, provision of system/s to capture and report incidents, issues, out of specification (OOS) or compliance events, serious breaches, change control and periodic audit of systems and sites (10).

The Control Site takes responsibility for the POCare platform once the product is ready for decentralized manufacturing, i.e., the technology transfer considered complete and successful (as described later).

### Gap analysis and risk assessment

The production process needs to be adapted to a process customizing an existing autologous CGT to decentralized POCare manufacturing requirements to provide products that are prepared both in a standardized/harmonized way and GMP compliant. To introduce a CGT to the POCare platform, both gap analysis and risk assessment are performed on the current production process and analytical methods for suitability. The focus is the end-product profile affected by the starting materials and variable parameters of the (decentralized) manufacturing/testing process. Quality by Design (QbD) methodology should be applied. QbD is a scientific, knowledge, risk-based framework aiming to establish product quality.

### Process development and approval of the GMP manufacturing process at POCare

**FIGURE 2:**
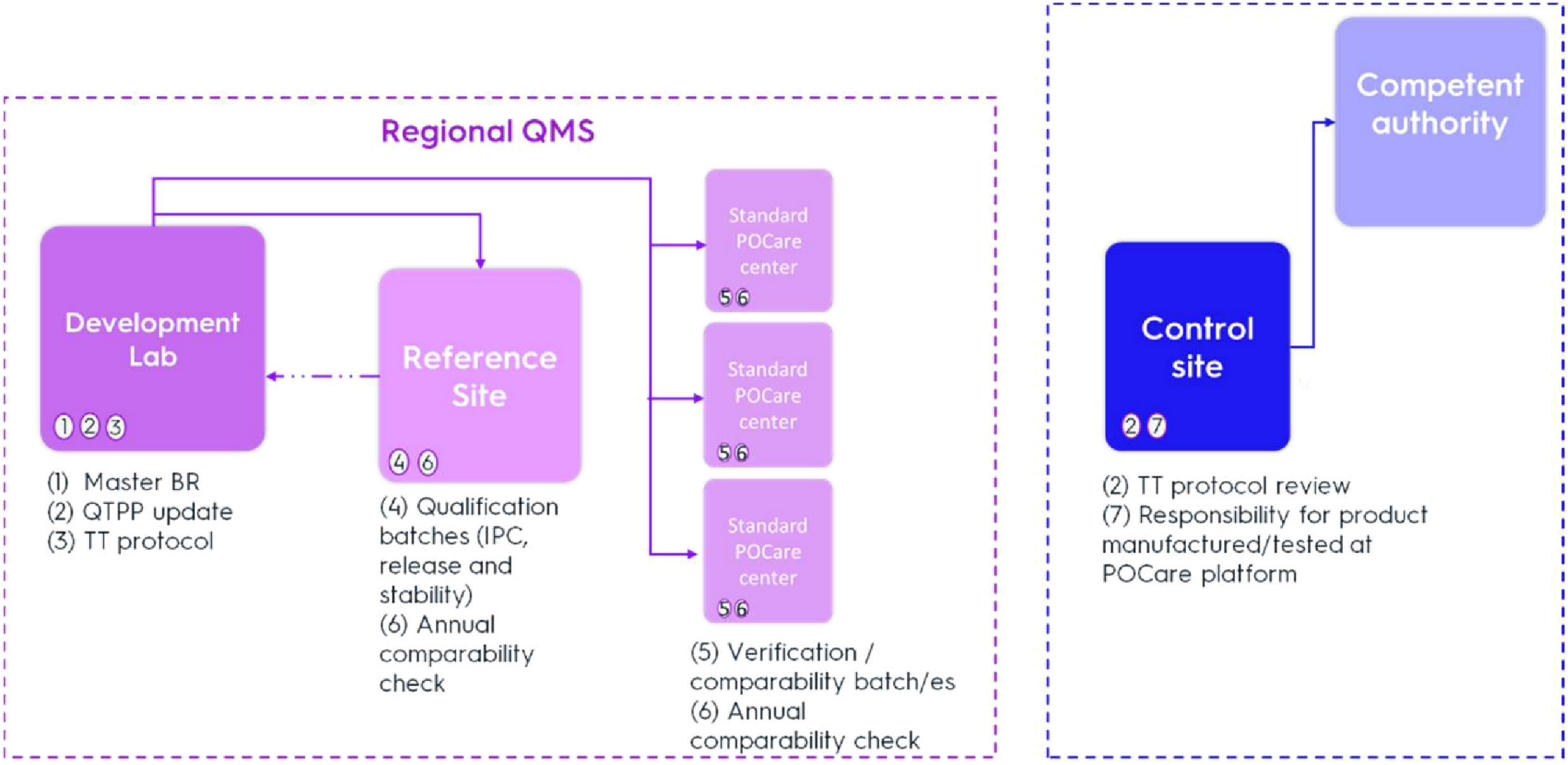
Regulatory steps of POCare process development and manufacturing. BR, batch record; QTPP, Quality Target Product Profile; TT, technology transfer; IPC, in process control. Numbers in brackets are explained in detail in the text.

The methodology starts with defining the quality, safety and efficacy characteristics of the desired final product, also known as the Quality Target Product Profile (QTPP) (Figure 2). In function of the QTPP, critical quality attributes (CQAs) that have a potential impact on product quality will be identified (30). The product and process knowledge acquired with Design of Experiments (DoEs), will be used to create a design space based on risk-based process analysis that supports the development process/testing studies (30). A process development proposal will be provided to product developer approval followed by a detailed development program (Figure 2).

After gap analysis, targeted process development will be initiated by the process development team introducing off-the-shelf closed/semi closed automated/semi-automated systems adjusting the manufacturing process/analytical methods to the POCare platform by reducing open processing as much as possible (Figure 2, step 1–3). As needed, customized integrated automated closed systems are introduced to ease scalability by further eliminating manual intervention as much as possible. Technologies such as digital quality management systems (QMS), electronic batch records, and integrated data management platforms represent critical enablers for decentralized models and should be implemented as much as possible.

Once the development product is considered suitable for POCare manufacturing and testing by the process development team, full-scale engineering batches are manufactured/tested at the development lab and a written report is issued including the suitability level of this product to POCare manufacturing. The product may be suitable for either early-stage (≤ 5 POCare centers, limited geographical extend) or late-stage decentralized manufacturing, meaning that the product is suitable for mass decentralized manufacturing at POCare centers around the globe (> 5 POCare centers).

This “end of process development” includes (a) the technology transfer scope, (b) the gap analysis/risk assessment identifying potential hazards in relation to POCare manufacturing/testing, and (c) the recommendation for the comparability that will be required once changes are introduced based on in-depth understanding of the specific product manufacturing process and related analytical tools. The QTPP is updated accordingly.

Once a CGT product is considered suitable for clinical grade manufacturing, a technology transfer (TT) of the manufacturing process and analytical methods to an assigned qualified *POCare center* will be performed. This POCare center will be defined as the “*Reference Site*” (Figure 2, step 4). The technology transfer protocol generated by the development team will be reviewed and approved by regional QA leader/s and *Control Site* team designee (Figure 2, step 2).

Three verification batches should be manufactured at the assigned *Reference site* (Figure 2, step 4), including extensive in process control (IPC), release testing and stability studies as determined and detailed in the “end of process development” report. The reference site will act as a point of comparison for all other POCare centers once initiated. The TT process will be accompanied and supervised by the regional QA leader and the “*Control Site*” team. The QTPP will be updated naming the “reference site” and addressing any issues identified during the TT. The QP at Control Site will be responsible for product release and interaction with competent authority (Figure 2, step 2 and 7). When identical automated systems and standardization have been established for manufacturing the number of qualification batches may be reduced. Platform interconnectivity of the necessary information technology applications should be established. As stated in Annex 15 (Qualification and Validation) of EU GMP Guidelines in section 5.20 “*an alternative number of batches may be justified taking into account whether standard methods of manufacture are used and whether similar products or processes are already used at the site*” (31).

For any additional POCare qualification (Figure 2, step 5), at least one verification/comparability batch should be manufactured including stability study (extent is based on the QTPP). The extent of comparability exercise depends on risk assessment, taking into consideration multiple factors that impact the manufacturing process/testing, POCare center and CGT nature. The “reference POCare site” acts as the reference standard for all POCare sites.

On an annual basis it is suggested to perform a “comparability check” procedure (Figure 2, step 6). This procedure should include analysis of both selected manufacturing steps and selected analytical method/s. The “reference site” and selected POCare centers will manufacture and test a product utilized form the same (shared) starting material to further establish continuous equivalence.

## Discussion

In conclusion, under the provision of GMP standards a regulatory framework can be established for decentralized manufacturing of cell or gene therapy products at or close to point of care. As a “living drug” the intrinsic variety of the patients’ starting material and the subsequent variability of the therapeutic product encompasses a situation similar to bone marrow transplantation or blood products which are unique singular products administered to patients. This fact represents a fundamental difference to pharmaceutical production of traditional drugs. In 2007, a clinical trial application designed to investigate a specific modality of hematopoietic stem cell transplantation (HSCT) in children and adolescents was submitted to authorities in Germany, Czech Republic and Austria. This clinical trial was focused on acute myeloid leukemia, with HSCT being the Investigational Medicinal Product (IMP). Newly introduced regulatory requirements asked for the IMP to be prepared in GMP conditions even though HSCT was already considered standard of care. In close collaboration with the German regulatory authority (Paul-Ehrlich Institute) a general IMP dossier (IMPD) was developed allowing for an individual preparation of HSCT investigational products according to GMP standards with a respective quality and safety profile (32). Since then a regulatory mindset for autologous therapeutic products has evolved considering the differences between classical pharmaceutical drug products and autologous individual patient-based cell therapies. In this situation, the process risk analysis must identify critical quality attributes (CQA) ensuring consistent quality of autologous cell therapy products.

If the expectations of decentralized manufacturing are realized either partially or in a more comprehensive manner by means of a high degree of automation and closed-system processing of multiple products in parallel, the result will be a marked reduction in operating costs for all steps of the process (33). Digitization represents one of the fundamental requirements for cost effective decentralized manufacturing. This includes critical enablers such as digital quality management systems, electronic batch records, and integrated data management platforms. We here propose a regulatory oversight mechanism based on a Control Site which will be the only establishment carrying the manufacturing authorization. Again, as much as possible digitization of processes and communication will support remote oversight. The Control Site will maintain the POCare Master File for the individual POCare GMP manufacturing sites. A standardized GMP manufacturing platform (e.g., deployable as mobile units and allowing quick expansion) and an overarching training platform should guarantee the quality standards in manufacturing by reducing hardware-related deviations and (manual) process variabilities. In January 2025, the UK government introduced a first-of-its-kind framework to make it easier to manufacture innovative medicines at POCare (11). The establishment of such a regulatory framework for POCare manufacturing will bring a range of benefits to patients, healthcare professionals and innovators. One important benefit is given in the timely and cost-efficient provision of products that need to be tested and released rapidly due to short shelf life, or because of patient’s rapidly declining health status. Furthermore, patients with inherited diseases may require a CGT, but the small overall number of patients with this disease might not build an acceptable business case for pharmaceutical development. In this situation, academic POCare centers can increase their flexibility in manufacturing capacity by a decentralized manufacturing approach with predefined deployable production units allowing quick set up of production. This may be specifically relevant for cell therapy products with limited stability, or which may lose some of their therapeutic activity by cryopreservation like Tumor Infiltrating Lymphocytes (TILs), Mesenchymal Stromal Cells (MSC), or CAR-T cells. Allogeneic products may also be manufactured in a smaller scale including patient specific manufacturing steps (directed manufacturing) benefiting from decentralized manufacturing.

The risk-based approach described here underpins a GMP approach for CGT products enabling product manufacturing “close to the patient” (POCare). The EMA GMP guideline for ATMP (18) already mentions flexibility regarding DCM and the use of automated equipment. CBER describes manufacturing units that can be deployed to multiple locations and POCare Manufacturing in proximity to patient care (15). Regulatory challenges must be tackled including questions of comparability of products manufactured at multiple sites and mechanisms for introduction of new sites within the current variation framework. A further challenge of the proposed framework includes logistical challenges like just in time availability of media. Again, a POCare network seems to be the best solution to tackle this challenge. The proposed QMS system is based on control sites where the qualified person is authorized to be responsible for decentralized manufacturing sites. The concept of “distributed” sites represents a logical and feasible solution to ensure both continued patient access to marketed products and robust development of future therapies (34). Collaboration between academic programs and industry could be jointly fruitful and cost-effective to rapidly bring to the field much needed therapies for patients in need or underserved areas (35), in particular by employing regional decentralized manufacturing centers with supply chain flexibility supporting clinical roll-out of cell therapies (3).

In summary, decentralized manufacturing has the potential to facilitate accessibility of cell and gene therapies. The proposed regulatory framework provides GMP control measures equivalent to those currently in place for medicinal products, ensuring that POCare products have appropriate quality, safety, and efficacy attributes. Clarity is needed on regulatory expectations, e.g., the demonstration of consistency and comparability, the central role of QP, and the Control Site as single point of contact for competent authorities.

## Data Availability

The original contributions presented in this study are included in this article/supplementary material, further inquiries can be directed to the corresponding author.
